# Urate-lowering therapy and kidney outcomes in patients with chronic kidney disease and hyperuricemia

**DOI:** 10.1038/s41392-025-02497-0

**Published:** 2025-12-09

**Authors:** Sheng Nie, Shiyu Zhou, Ruixuan Chen, Lantian Li, Yinfang Sun, Jiao Liu, Luhua Jin, Xian Shao, Mingzhen Pang, Licong Su, Fan Luo, Xin Xu, Fan Fan Hou

**Affiliations:** State Key Laboratory of Multiorgan Injury Prevention and Treatment, Guangdong Provincial Key Laboratory of Renal Failure Research, Division of Nephrology, National Clinical Research Center for Kidney Disease, Guangdong Provincial Institute of Nephrology, Nanfang Hospital, Southern Medical University, Guangzhou, China

**Keywords:** Kidney diseases, Kidney diseases

## Abstract

Hyperuricemia is considered a modifiable risk factor for the development and progression of chronic kidney disease (CKD). There remains controversy over the effects of urate-lowering therapy (ULT) on kidney outcomes in patients with CKD and hyperuricemia. We conducted a cohort study using a sequential target trial emulation framework to evaluate the composite kidney outcomes in patients with CKD and hyperuricemia initiating ULT versus supportive care alone (control). A total of 269,831 eligible person trials (56,936 unique persons) with CKD and hyperuricemia who had received supportive care were included from the China Renal Data System database. The primary outcome was a composite kidney outcome defined as a greater than 40% decline in the estimated GFR or end-stage kidney disease (ESKD). The 3-year cumulative incidence rates of the composite kidney outcomes were 19.69% and 23.22% in the ULT group and the control group, respectively, with a risk difference of −3.53% (95% CI, −5.25% to −1.94%). The estimated 3-year risk differences for ESKD, all-cause mortality, and cardiovascular mortality were −1.88% (−3.28% to −0.45%), −2.25% (−3.02% to −1.51%), and −0.69% (−1.33% to −0.05%), respectively, all of which favor the ULT group. The estimates from the subgroup and sensitivity analyses were consistent with those from the primary analysis. Thus, ULT is associated with a significantly lower risk of kidney disease progression and mortality in patients with stage 3 or higher CKD and hyperuricemia. Large randomized clinical trials with refined designs are needed to assess the effect of ULT in these patients.

## Introduction

Chronic kidney disease (CKD) represents one of the most critical and escalating non-communicable diseases globally, affecting 8–14% of adults worldwide.^1–3^ Patients with CKD face a markedly increased risk for end-stage kidney disease (ESKD) as well as a heightened susceptibility to major cardiovascular events.^4^ This confluence of complications leads to a profoundly worsened prognosis across all stages of the disease. It accounts for approximately 2.6 million deaths annually, of which 0.96 million deaths are due to ESKD.^5^ Forecasting study, including the Global Burden of Disease (GBD) Study 2021, predicted that CKD will become the 10th leading cause of death in the near future.^6^ Recognizing this impending catastrophe, the World Health Organization (WHO) has initiated a global petition to formally establish CKD as a global health priority, underscoring the urgency of effective intervention strategies.

Hyperuricemia, characterized by elevated serum uric acid levels, is a common and modifiable comorbidity among patients with CKD. According to the United States National Health and Nutrition Examination Survey spanning 1999–2018, the prevalence of hyperuricemia is disproportionately high in the CKD population, affecting 24% of these patients compared to 11.9% in the general population.^7,8^ Furthermore, the prevalence intensifies dramatically with advancing kidney disease stage, demonstrating a nearly ten-fold higher rate in stages 3–5 than in stage 1.^9^ Observational data also link hyperuricemia not only to the development and accelerated progression of CKD but also to increased risks for major cardiovascular events and all-cause mortality in this vulnerable patient cohort.^10–12^ However, the role of increased serum uric acid levels in the deterioration of kidney function remains obscure. Increased uric acid levels may also result from reduced renal excretion, diuretic use, or increased oxidative stress.^13^ This raises a critical clinical question: is uric acid merely a biomarker of declining renal function, with meaningless benefit to treat, or does it serve as a modifiable target that contributes to CKD progression, which will improve the prognosis of CKD patients with good management? If uric acid is primarily an innocent marker of reduced renal clearance, then urate-lowering therapy (ULT) may be clinically meaningless for slowing disease progression. Conversely, if it is an active risk factor, targeted management of hyperuricemia could significantly improve the prognosis for CKD patients.

Despite the strong observational links, no adequately powered evidence has proved the beneficial effects of ULT in patients with CKD. Prior clinical trials, such as those conducted by Siu et al. and Goicoechea et al., provided initial supportive evidence, suggesting that allopurinol might slow the progression of renal disease and reduce the risk for cardiovascular disease.^14,15^ These earlier studies employed relatively hard outcomes, such as cardiovascular events or CKD progression defined by a greater than 40% increase in serum creatinine. However, three recently reported randomized controlled trials (RCTs) do not find the benefit of ULT in slowing kidney function loss in diverse patient groups, including those with type 1 diabetes mellitus, progressed CKD without gout, or CKD and asymptomatic hyperuricemia.^16–18^ The collective body of this evidence is marked by population heterogeneity, enrolling participants with broad age ranges (e.g., 40–70 years) and widely varying baseline kidney function (estimated glomerular filtration rate [eGFR] levels spanning 15–59 ml/min/1.73 m^2^ to 40–99 ml/min/1.73 m^2^). Furthermore, some trials included participants with normal serum uric acid levels. The endpoints used across these trials were predominantly surrogate outcomes, such as the absolute change or slope of the eGFR. The current Kidney Disease Improving Global Outcome guideline prudently recommends against the routine use of urate-lowering agents in CKD patients with asymptomatic hyperuricemia for the sole purpose of slowing disease progression, admitting the low certainty and discordance of these results.^19^ The clinical utility of ULT among patients with CKD and hyperuricemia in real-world practice remains unresolved.^20,21^ This inconsistency and of RCTs necessitate evidence from real-world data to capture effectiveness across diverse patient groups, based on management in routine clinical settings to evaluate the true effectiveness of ULT against definitive, long-term clinical outcomes.

To address this critical knowledge gap and provide robust evidence, we designed the current study to evaluate the real-world effectiveness of ULT in slowing CKD progression. We aimed to emulate a target trial using a large, retrospective cohort from the China Renal Data System (CRDS), including 28 regional medical centers across China. This novel methodology allows us to rigorously compare treatment effects while minimizing the biases inherent in observational data. Ultimately, the findings have the potential to add real-world evidence on the effect of ULT among patients with CKD and asymptomatic hyperuricemia.

## Results

### Study population and baseline characteristics

**Fig. 1:**
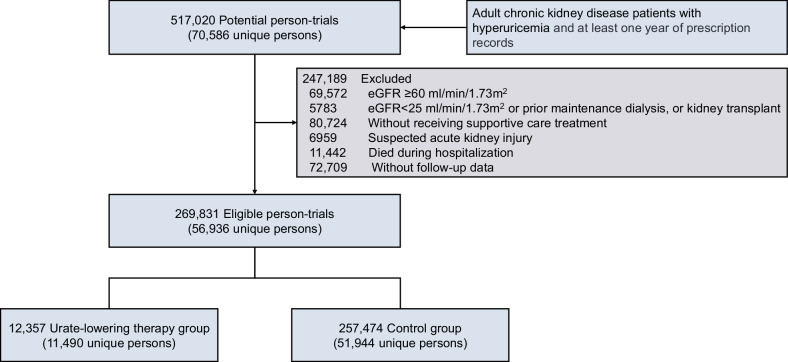
Flowchart of the study population. ^*^Supportive care, including treatment for hypertension, dyslipidemia, anemia, chronic kidney disease-mineral and bone disorders, hyperkalemia, acidosis, etc., in patients with chronic kidney disease

Following the sequential target trial framework (Supplementary Table 1), 517,020 potential person trials (70,586 unique persons) were identified, of which 269,831 (56,936 unique persons) met all the eligibility criteria and were included in our emulated target trial (Fig. 1). At enrollment, 12,357 and 257,474 person trials were assigned to the ULT group and the control group, respectively. A summary of the emulated trials among patients in the urate-lowering therapy group versus the control group is shown in Supplementary Table 2. Overall, 141,900 (52.6%) patients were male, the median age was 71 (IQR, 61–80) years, and the median serum uric acid level and eGFR at enrollment were 8.1 (IQR, 7.3–9.2) mg/dL and 46 (38–53) ml/min/1.73 m^2^, respectively (Table 1). The median number of serum uric acid measurements per patient was 4 (IQR 2–9), and the median number of creatinine measurements was 4 (IQR 2–10) during follow-up. Compared with the control group, the ULT group had a greater proportion of males (62.7% vs 52.1%), a greater baseline serum uric acid level (9.4 vs. 8.0 mg/dL), and a lower eGFR (43 vs. 46 ml/min/1.73 m^2^). Gout was more prevalent in the ULT group than in the control group (19.9% vs. 8.9%). The serum uric acid level in the ULT group was slightly lower than that in the control group and remained stable during follow-up (Supplementary Fig. 1). A comparable median of 5 (IQR 2–11) serum creatinine measurements was found in both groups throughout the follow-up period.

**Table 1:** The baseline characteristics of the person trials in the emulated target trial

Characteristics	Total (269,831 person trials)	Urate-lowering therapy group (12,357 person trials)	Control group (257,474 person trials)	Missing proportion (%)
Age, year	71 (61–80)	70 (60–79)	71 (61–80)	0
Sex, Male (%)	141,900 (52.6)	7744 (62.7)	134,156 (52.1)	0
Hosplization (%)	80,363 (29.8)	7355 (59.5)	73,008 (28.4)	0
Number of hospitalizations in the year	1 (0–2)	1 (0–1)	1 (0–2)	0
Intensive care unit (%)	2307 (0.9)	241 (2.0)	2066 (0.8)	0
Number of Intensive care units in the year	0 (0–0)	0 (0–0)	0 (0–0)	0
Body mass index, kg/m^2^	21.9 (21.1–25.0)	21.9 (21.2–25.4)	21.9 (21.0–25.0)	54.6
SBP, mmHg	131 (120–145)	133 (120–147)	131 (120–145)	65.9
DBP, mmHg	76 (68–83)	76 (69–86)	75 (68–83)	65.9
Surgery (%)	30,876 (11.4)	1227 (9.9)	29,649 (11.5)	0
Calendar years of patient inclusion (%)				0
2012	2407 (0.9)	63 (0.5)	2344 (0.9)	
2013	3459 (1.3)	108 (0.9)	3351 (1.3)	
2014	10,455 (3.9)	374 (3.0)	10,081 (3.9)	
2015	20,095 (7.4)	670 (5.4)	19,425 (7.5)	
2016	31,435 (11.6)	1297 (10.5)	30,138 (11.7)	
2017	37,597 (13.9)	1486 (12.0)	36,111 (14.0)	
2018	42,870 (15.9)	2061 (16.7)	40,809 (15.8)	
2019	42,452 (15.7)	2036 (16.5)	40,416 (15.7)	
2020	32,775 (12.1)	1832 (14.8)	30,943 (12.0)	
2021	28,263 (10.5)	1601 (13.0)	26,662 (10.4)	
**Laboratory parameters**				
Serum uric acid, mg/dL	8.1 (7.3–9.2)	9.4 (8.4–10.8)	8.0 (7.2–9.1)	0
Number of serum creatinine tests within 6 months	2 (1–4)	2 (1–4)	2 (1–4)	0
Serum creatinine, μmol/L	123 (106–146)	132 (114–157)	123 (106–145)	0
eGFR, ml/min/1.73 m^2^	46 (38–53)	43 (35–51)	46 (38–53)	0
<30	22,776 (8.4)	1398 (11.3)	21,378 (8.3)	
30–44	104,291 (38.7)	5455 (44.1)	98,836 (38.4)	
≥45	142,764 (52.9)	5504 (44.5)	137,260 (53.3)	
UACR, mg/g	17 (11–200)	16 (11–200)	17 (11–200)	21.3
<30	120,824 (44.8)	6064 (49.1)	114,760 (44.6)	
30–299	51,607 (19.1)	2458 (19.9)	49,149 (19.1)	
≥300	39,857 (14.8)	2004 (16.2)	37,853 (14.7)	
Unknown	57,543 (21.3)	1831 (14.8)	55,712 (21.6)	
Triglyceride, mmol/L	1.4 (1–2.1)	1.5 (1.0–2.3)	1.4 (1.0–2.1)	18.9
Total cholesterol, mmol/L	4.5 (3.6–5.4)	4.4 (3.6–5.4)	4.5 (3.6–5.4)	16.1
LDL-C, mmol/L	2.5 (1.9–3.3)	2.5 (1.9–3.2)	2.5 (1.9–3.3)	22.4
Serum albumin, g/L	40 (36–44)	40 (36–44)	40 (36–44)	7.9
Hemoglobin, g/L	122 (108–136)	124 (109–139)	122 (107–136)	11.3
Glycated hemoglobin level, %	6.4 (5.8–7.5)	6.3 (5.7–7.2)	6.4 (5.8–7.5)	70.2
**Comorbidity (%)**				0
Charlson score	6 (4–8)	5 (4–7)	6 (4–8)	
Gout	25,482 (9.4)	2453 (19.9)	23,029 (8.9)	
Kidney stone	53,393 (19.8)	2266 (18.3)	51,127 (19.9)	
Hypertension	172,181 (63.8)	7467 (60.4)	164,714 (64.0)	
Diabetes	92,281 (34.2)	3620 (29.3)	88,661 (34.4)	
Myocardial infarction	16,931 (6.3)	725 (5.9)	16,206 (6.3)	
Ischemic heart disease	82,503 (30.6)	3510 (28.4)	78,993 (30.7)	
PVD	68,380 (25.3)	2702 (21.9)	65,678 (25.5)	
Stroke	67,640 (25.1)	2847 (23.0)	64,793 (25.2)	
Heart failure	64,789 (24.0)	2985 (24.2)	61,804 (24.0)	
Cancer	48,293 (17.9)	1188 (9.6)	47,105 (18.3)	
Proteinuria	115,234 (42.7)	5184 (42.0)	110,050 (42.7)	
**Comedications (%)**				0
Number of medications in 3 months	10 (3–17)	13 (7–19)	10 (3–17)	
RASi	160,873 (59.6)	8124 (65.7)	152,749 (59.3)	
Statins	148,369 (55.0)	7558 (61.2)	140,811 (54.7)	
Diuretics	137,896 (51.1)	6735 (54.5)	131,161 (50.9)	
Calcium channel blockers	153,631 (56.9)	7391 (59.8)	146,240 (56.8)	
SGLT-2 inhibitors	5671 (2.1)	386 (3.1)	5285 (2.1)	

*IQR* interquartile range, *DBP* diastolic blood pressure, *LDL-C* low-density lipoprotein cholesterol, *PVD* peripheral vascular disease, *SBP* systolic blood pressure, *SGLT-2* sodium-glucose cotransporter 2, *RASI* retin angiotensin system inhibitor, *UACR* urine albumin-creatinine ratio

### Primary outcome

**Fig. 2:**
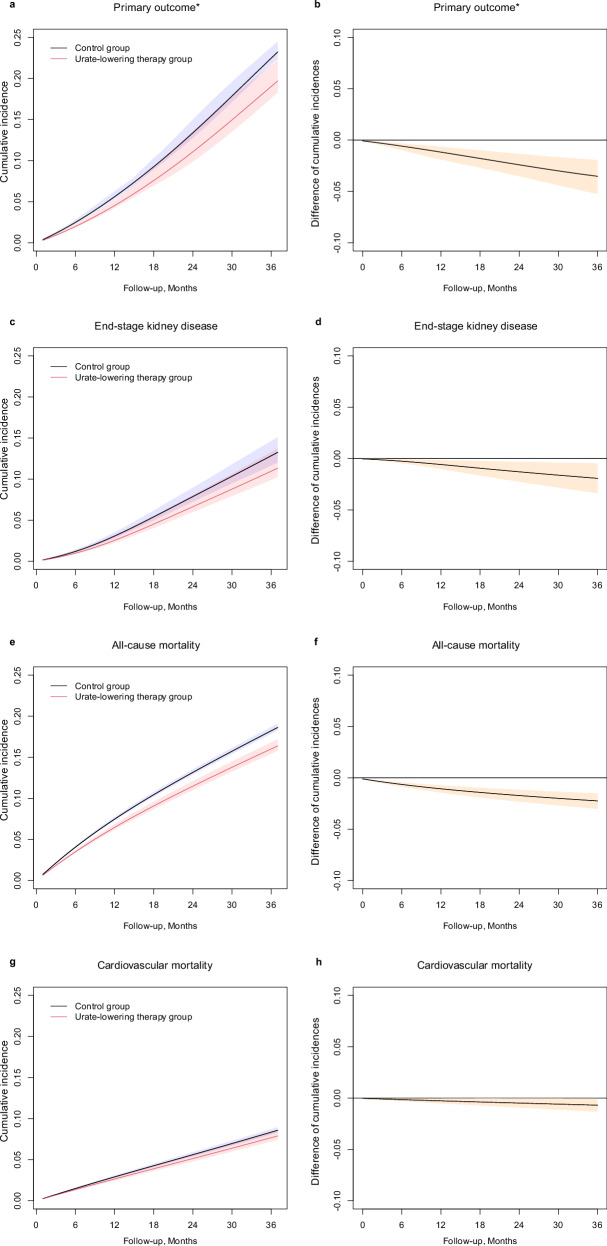
Three-year cumulative incidence rates and risk differences between the urate-lowering therapy group and the control group. ^*^Primary outcome was a composite of a 40% eGFR decline from baseline or end-stage kidney disease. Adjusted cumulative incidence of the primary outcome (**a**) and the corresponding risk difference (**b**) over time between the urate-lowering therapy group and the control group. Adjusted cumulative incidence of the end-stage kidney disease (**c**) and the corresponding risk difference (**d**) over time between the urate-lowering therapy group and the control group. Adjusted cumulative incidence of all-cause mortality (**e**) and the corresponding risk difference (**f**) over time between the urate-lowering therapy group and the control group. Adjusted cumulative incidence of cardiovascular mortality (**g**) and the corresponding risk difference (**h**) over time between the urate-lowering therapy group and the control group

During follow-up, a total of 927 and 4456 composite kidney events occurred in the ULT group and the control group, respectively. The curves of the adjusted cumulative incidences stratified by the two treatment groups are presented in Fig. 2a. In the intention-to-treat analysis, the adjusted cumulative incidence rates at the end of 3 years were 19.69% (95% CI, 18.30%–22.18%) in the ULT group and 23.22% (95% CI, 22.34%–24.49%) in the control group, with an estimated risk difference of −3.53% (95% CI, −5.25% to −1.94%) (Table 2 and Fig. 2b). In the per-protocol analysis (Supplementary Table 3), the estimated 3-year cumulative incidence rates were 16.62% and 22.84%, respectively, in the ULT group and the control group, with a risk difference of −6.22% (CI, −10.51% to −2.10%).

**Table 2:** Three-year cumulative incidence rates and risk differences among patients receiving urate-lowering therapy and control patients

Study outcome	No. of events/100 PY	Cumulative incidence, % (95% CI)	Risk difference, % (95% CI)
Primary outcome^a^			
Control	6.7	23.22 (22.34–24.49)	Reference
Urate-lowering therapy	7.2	19.69 (18.30–22.18)	−3.53 (−5.25 to −1.94)
Secondary outcomes			
End-stage kidney disease			
Control	3.9	12.84 (11.65–14.64)	Reference
Urate-lowering therapy	4.2	10.96 (9.90–13.30)	−1.88 (−3.28 to −0.45)
All-cause mortality			
Control	6.5	18.63 (18.27–19.09)	Reference
Urate-lowering therapy	6.0	16.38 (15.79–17.19)	−2.25 (−3.02 to −1.51)
Cardiovascular mortality			
Control	2.8	8.58 (8.29–8.95)	Reference
Urate-lowering therapy	3.1	7.89 (7.41–8.60)	−0.69 (−1.33 to −0.05)

*PY* person-year

^a^Primary outcome was a composite of a 40% eGFR decline from baseline or end-stage kidney disease

**Fig. 3:**
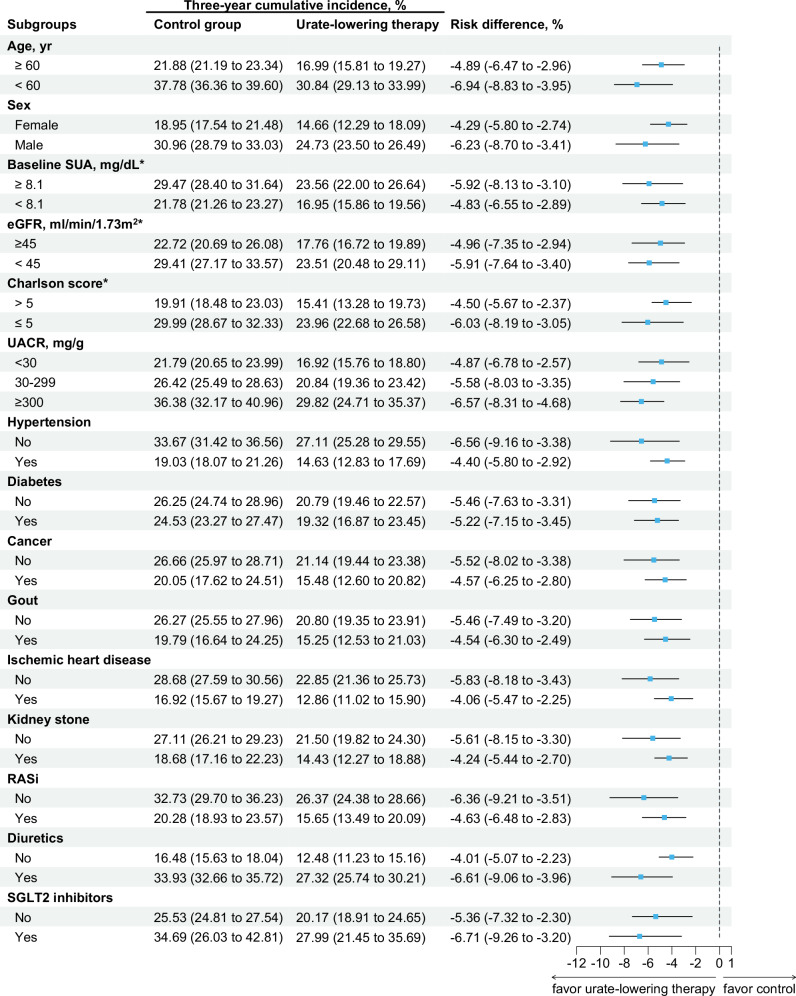
Subgroup analysis for the 3-year risk difference between the urate-lowering therapy group and the control group for the primary outcome. RASi renin angiotensin system inhibitor, SGLT-2 sodium-glucose cotransporter 2, UACR urine albumin-creatinine ratio. ^*^ Subgroup defined by the median at baseline

In the subgroup analysis, a lower 3-year risk favoring the ULT group was consistently observed among the subgroups (Fig. 3). Notably, a greater cumulative incidence of composite kidney outcomes and a greater treatment effect were observed in the subgroup with a urine albumin-creatinine ratio ≥300 mg/g than in the subgroup with a urine albumin-creatinine ratio <300 mg/g. No significant heterogeneity was observed across other prespecified subgroups.

### End-stage kidney disease, mortality, and safety outcomes

In the analyses of secondary outcomes (Table 2), the adjusted 3-year cumulative incidence rates of ESKD were 10.96% and 12.84% in the ULT group and the control group, respectively, with a risk difference of −1.88% (95% CI, −3.28 to −0.45) compared with the ULT group (Table 2 and Fig. 2c, d). Over 3 years of follow-up, 185 deaths occurred in the ULT group, whereas 3030 deaths occurred in the control group. The adjusted 3-year cumulative incidence rates of all-cause mortality were 16.38% and 18.63%, respectively, in the ULT group and the control group, with a risk difference of –2.25% (95% CI, −3.02% to −1.51%), favoring the ULT group (Table 2 and Fig. 2e, f). A similar trend of treatment effects was observed for cardiovascular mortality (Table 2 and Fig. 2g, h), with an estimated risk difference of −0.69% (95% CI, −1.33% to −0.05%). For safety outcomes (Supplementary Table 4), there was no significant difference between patients in the ULT group and those in the control group (risk difference, 0.07% [95% CI, −0.24% to 0.41%]).

### Sensitivity analysis

No associations were found between ULT initiation and the negative outcome control (Supplementary Table 4). We estimated the treatment effects of allopurinol, febuxostat, and benzbromarone in separate emulated target trials, and similar treatment effects on the composite kidney outcomes were observed for the three urate-lowering drugs. (Supplementary Table 5). Similar treatment effects of ULT were also observed in the emulated target trials using 1:1 propensity score-matched person trials (Supplementary Tables 6 and 7), accounting for the competing risk of all-cause death (Supplementary Table 8), and the trial excluding patients with gout at enrollment (Supplementary Tables 9 and 10).

## Discussion

In this study of 56,936 eligible adults with CKD and hyperuricemia, we emulated a sequence of hypothetical randomized trials to evaluate the real-world efficacy of ULT in slowing the progression of kidney disease. We found that the initiation of ULT was associated with a significantly lower risk of kidney disease progression. Treatment effects favoring ULT were consistently observed among subgroups stratified by age, sex, baseline levels of serum uric acid and eGFR, and the presence of hypertension, diabetes, and gout. Sensitivity analyses yielded similar results, suggesting the robustness of the findings. ULT was also associated with a lower risk of all-cause mortality and cardiovascular mortality, further strengthening the clinical significance associated with ULT.

Hyperuricemia is common in CKD patients and could be a consequence of reduced urinary excretion of uric acid. As a consequence, the prevalence of hyperuricemia increases from 11% among subjects with normal kidney function to 60% in patients with CKD and 80% among those with stage 4 CKD.^22^ It is therefore important to understand whether ULT affects kidney and cardiovascular outcomes, especially in patients with CKD and hyperuricemia but without gout. To date, there remains controversy over the role of uric acid in adverse outcomes among these patients.

Evidence from epidemiologic studies,^11,23^ experimental studies^24,25^, and clinical trials^26^ has supported the hypothesis that hyperuricemia is a modifiable risk factor for CKD progression. However, two recent trials, known as Prevention of Early Renal Loss (PERL) in diabetes^16^ and the Controlled Trial of Slowing of Kidney Disease Progression from the Inhibition of Xanthine Oxidase (CKD-FIX)^17^, reported no benefit of allopurinol in slowing kidney progression in patients with CKD and asymptomatic hyperuricemia. This has led several groups to suggest that asymptomatic hyperuricemia in CKD patients is benign and should not be treated.^27,28^ Our study, through a large sample target trial, revealed that uric acid-lowering drugs were associated with lower risks of kidney disease progression and mortality in the real world, suggesting a countering viewpoint.

Several key differences in the study design might contribute to these inconsistent findings. First, our study enrolled over 56,000 CKD patients at high risk of progression (CKD stage 3 or higher). All patients had hyperuricemia, with a mean uric acid level of 8.1 mg/dL. In comparison, the PERL and CKD-FIX trials included large numbers of patients with normal levels of serum uric acid. Normal uric acid levels are not expected to increase the risk of CKD progression.^20^ The inclusion of lower-risk patients has resulted in lower event rates and inferior statistical power in trials on the progression of CKD.^29^ Second, our study used a composite endpoint of a greater than 40% decline in the eGFR or ESKD as the hard kidney outcome, whereas most published CKD progression trials, including PERL and CKD-FIX, used the eGFR slope as a surrogate for kidney failure. Although it is reasonable to believe that a reduction in the rate of eGFR decline in the long term will reduce the risk for kidney failure, it is uncertain how to reliably translate the estimated change in the eGFR slope into a reduction in the risk of kidney failure, as eGFR changes may occur acutely or in a nonlinear fashion over time.^30^ Third, in our study, after accounting for the competing risk of all-cause death, the 3-year risks of the composite kidney outcome were 19.09% and 22.28% in the urate-lowering and control groups, respectively, representing a 14.32% relative attenuation in the risk. However, both the PERL and CKD-FIX trials, with moderate sample sizes of 363 and 530, respectively, were designed to detect a 20–30% attenuation in the GFR slope compared with that of the control. The statistical power of these two trials was further limited by the low recruitment rate (60% in the CKD-FIX trial) and high rate of loss to follow-up (20% in the PERL trial). Therefore, the PERL and CKD-FIX trials may not have adequate power to detect the effect size. Consistent with our results, two recent studies reported that lowering the level of serum urate to below 6 mg/dl in patients with gout attenuated the decline in kidney function^31^ and reduced the risk of kidney failure.^32^

In this study, ULT was associated with 2.25% and 0.69% lower risks of all-cause and cardiovascular mortality, respectively, than were the controls. Consistent with our findings, previous population studies also reported an association between ULT and lower mortality in patients with hyperuricemia and CKD, although some of those studies failed to address time-related bias.^33^ In a cohort study of 5277 propensity score-matched pairs of allopurinol initiators and noninitiators from the Health Improvement Network UK primary care database, allopurinol initiation among patients with gout and CKD was associated with lower mortality (hazard ratio, 0.85; 95% confidence interval, 0.77–0.93) than noninitiation in nonallopurinol users.^34^ Furthermore, among allopurinol initiators, those who had achieved a target level of uric acid within 1 year had a lower risk of 5-year mortality (risk difference −3.36%; 95% CI, −7.33% to −0.68%) than those who had not.

Our sensitivity analysis, which compared three urate-lowering drugs against a control group, revealed that both allopurinol and benzbromarone were associated with a lower risk of the composite kidney outcome, whereas febuxostat showed a similar but nonsignificant trend. This lack of significance for febuxostat may stem from its later market introduction, leading to insufficient follow-up time and limited statistical power. The three drugs differ in their mechanisms of action, and there is currently no direct evidence to suggest which offers superior kidney protection. Benzbromarone, for example, has been linked to potential effects beyond its urate-lowering action, such as the ability to scavenge radicals and reduce oxidative stress, which warrants further investigation.^35^ Future head-to-head comparative studies are needed to guide optimal drug selection among these agents.

Despite being the largest clinical real-world study evaluating ULT in a CKD population reported to date, our study has several limitations. First, it is an observational study by nature, in which residual and unmeasured confounding factors cannot be ruled out. Specifically, the absence of information regarding lifestyle and dietary factors may introduce residual confounding factors. Nevertheless, we have made a great effort to minimize potential confounding and selection bias by using a new user and sequentially emulating the target trial design with vigorous adjustment for both the time-fixed and the time-varying covariates. We have also performed several sensitivity analyses to evaluate the robustness of our findings. Second, treatment assignment in our analysis was based on prescription data from the participating hospitals. The medication actually taken by the patients, as well as medications over the counter or prescriptions outside of the participating hospitals, were not available, which may cause misclassification of treatment assignment and per-protocol determination. Third, we did not perform a dose‒response analysis in the present study, as more presumptions are required for such an analysis. Finally, our study population is predominantly Chinese. Verification of our findings in real-world studies from other populations is needed. In this real-world clinical study, ULT was associated with a significantly lower risk of kidney disease progression and mortality in patients with stage 3 or higher CKD and hyperuricemia, suggesting that treating hyperuricemia may be beneficial for patients with CKD. Larger clinical trials with refined designs are needed to assess the effects of ULTs on outcomes in these patients.

## Materials and methods

### Data source

The CRDS was a joint initiative of the National Clinical Research Center for Kidney Disease and the China Center for Disease Control and Prevention (CDC) in 2018. Currently, this database covers 27 million individuals from 28 tertiary hospitals across China. The patient-level data from each center were cleaned, standardized, anonymized, and pooled at the CRDS data center located at the National Clinical Research Center of Kidney Disease in Guangzhou. The database includes both inpatient and outpatient data, including demographic information, vital signs, diagnoses, laboratory measurements, prescriptions, and medical records.^36,37^ Death data, including date and cause of death, were obtained by linking to the Cause of Death Reporting System in the China CDC.^38^

The study was approved by the Medical Ethics Committee of Nanfang Hospital, Southern Medical University (approval no. NFEC­201905-K10 and NFEC-2023-409), and the requirement for informed consent was waived.

### Study design

We followed the framework proposed by Hernán and Robins^39,40^ to emulate a sequence of nested target trials that enrolled adults with CKD and hyperuricemia in each calendar month between January 2012 and December 2022 and assessed the intention-to-treat efficacy of initiating ULT in slowing the decline in kidney function. We required the initiation of ULT, defined as having no such prescription during the 1-year period before enrollment. The sequential nested target trial emulation is chosen for mitigating the immortal-time bias and handling this dynamic nature. The key elements of the target and emulated trials are summarized in Supplementary Table 1 and detailed in the following sections.

### Eligibility criteria

In the target trial of each calendar month, we first identified adults (≥18 years old) with CKD and hyperuricemia (serum uric acid >7 mg/dL in men or 6 mg/dL in women) who had received supportive care and had at least 1 year of medication prescription records. CKD was diagnosed if any one of the following three criteria was met: (1) an eGFR of less than 60 mL/min/1.73 m^2^ persisted for more than 90 days; (2) persistent proteinuria for over 90 days; or (3) *International Classification of Diseases, 10th Revision* codes of N02–N08, N11, N15.0, N18–N19, N25–N27, N28.818, N39.1, I12–I13, I15.102–I15.103, E10.2, E11.2, E12.2, E13.2, and E14.2. The exclusion criteria were as follows: (1) use of ULT within 1 year prior to enrollment; (2) an eGFR at enrollment of more than 60 or less than 25 ml/min/1.73 m^2^; (3) receiving maintenance dialysis or kidney transplant at enrollment; (4) not receiving supportive care treatment; and (5) suspected acute kidney injury (defined as the fluctuation of serum creatinine >1.5 times) within 3 months prior to enrollment. Supportive care, including treatment for hypertension, dyslipidemia, anemia, CKD-mineral and bone disorders, hyperkalemia, acidosis, etc., is recommended for patients with CKD.^19^

### Treatment assignment

A total of 132 consecutive trials, with each calendar month as the enrollment period, were carried out from January 2012 to December 2022. In each trial, eligible patients were assigned to the ULT group if they initiated therapy during the enrollment month; otherwise, they were assigned to the control group. The treatment assignment was not changed during the trial, following an intention-to-treat principle.

### Outcome assessment

The primary outcome was the composite kidney outcome, which was defined as either a sustained >40% decline in the eGFR relative to the baseline value at inclusion for at least 90 days or progression to ESKD. The secondary outcomes included ESKD, all-cause mortality, and cardiovascular mortality. ESKD was defined as an eGFR of less than 15 ml/min/1.73 m^2^ (sustained for at least 90 days), kidney transplant, or maintenance dialysis. Cardiovascular mortality was defined as death according to the *International Classification of Diseases, 10th Revision* (ICD-10) codes I00–I99 extracted from the mortality registry. Safety outcomes, including cutaneous reactions (L27.0–L27.1, L27.8–L27.9, T78.4, L50.0–L50.3, L50.8–L50.9, L51.0–L51.2, L51.8–L51.9, D72.102, L08.0, T88.7, R21, M33.1), hypersensitivity (T50.400), or hepatotoxicity (K71), are defined as the ICD-10 code. Negative outcome control was used to assess the presence of potential spurious biases from the statistical analyses. We examined the association between ULT use and the negative outcome control of gastritis and duodenitis (ICD-10 codes, K29). Follow-up started at each eligible month (target trial index date) and continued until the occurrence of study outcomes, death, or the end of the study period (December 31, 2022), whichever occurred first.

### Comorbidities, laboratory assays, and medications at enrollment

The presence of comorbidities at enrollment was identified by the International Classification of Diseases-10 (ICD-10) (Supplementary Table 11). Medication was defined as any prescription of medication within 365 days. Laboratory measurements at enrollment were defined as the most recent measurement within 90 days prior to enrollment. The eGFR was calculated from the serum creatinine level via the 2021 CKD-EPI creatinine equation.^41^ For patients with a missing urine albumin-to-creatinine ratio, we will convert the urine protein-to-creatinine ratio or dipstick protein to the urine albumin-to-creatinine ratio, if available.^42^

### Statistical analysis

The target trial causal contrasts of interest were the marginal intention-to-treat effects of ULT on the 3-year cumulative incidence of the outcome events. This marginal effect is defined as the difference in the cumulative incidences (risk differences) between two hypothetical scenarios: one where all participants in the target population were assigned to ULT and another where none were assigned.

We estimated the intention-to-treat effects via a marginal structural model for the sequence of emulated trials.^43^ The model was fitted by pooled discrete-time logistic regressions using a stabilized inverse probability of censoring weighting. For the primary outcome, a patient was censored at 30 days after the last follow-up with eGFR measurement, death, or 60 months, whichever came first. We adjusted the logistic model using the covariates at enrollment, including age, sex, blood pressure, eGFR, the urine albumin-creatinine ratio, the serum levels of uric acid, triglyceride, total cholesterol, low-density lipoprotein cholesterol, glycated hemoglobin A1c, albumin, and hemoglobin, the presence of comorbidities (gout, kidney stone, diabetes, ischemic heart disease, hypertension, cancer, myocardial infarction, peripheral vascular disease, stroke, ischemic heart disease, and heart failure), the need for hospitalization (binary variable), the number of hospitalizations in the year, intensive care unit admission (binary variable), the number of intensive care units in the year, surgery, and medication use (total number of medications in 3 months, renin angiotensin system inhibitors, statins, diuretics, calcium channel blockers, and sodium-glucose cotransporter 2 inhibitors). The model included both fixed baseline variables (e.g., sex and baseline laboratory findings, baseline comorbidities, and baseline medications) and time-varying variables (e.g., age, body mass index, hospitalization, intensive care unit admission, surgery, laboratory measurements, comorbidities, and medications). We calculated the censoring weights at each discrete time via logistic regression models that included the covariates at enrollment as well as the time-varying covariates. The last observation carried forward method was used to fill the missing covariates longitudinally. For each covariate with missing data at baseline, we applied median imputation and introduced an indicator variable for missing data.

In addition, we also estimated the per-protocol marginal effects of ULT. In the per-protocol analysis, follow-up data were artificially censored at 90 days after treatment discontinuation or upon treatment switching, and the inverse probability of treatment weight at each discrete time was estimated separately for each treatment group and used to adjust for selection bias that could otherwise arise from such censoring.

We performed analyses on the primary outcome in the subgroups stratified by age (≥60 and <60 years), sex, median serum uric acid at baseline (≥8.1 and <8.1 mg/dL), baseline eGFR (≥45 and <45 mL/min/1.73 m^2^), median Charlson’s comorbidity score (>5 and ≤5), baseline urine albumin-creatinine ratio (<30, 30–299, and ≥300 mg/g), history of hypertension, diabetes, ischemic heart disease, cancer, gout, kidney stones, use of renin angiotensin system inhibitors, diuretics, and sodium-glucose cotransporter 2 inhibitors.

We performed four sensitivity analyses to assess the robustness of our estimates. First, we estimated the marginal effect of each individual drug (allopurinol, febuxostat, or benzbromarone) separately. Second, we estimated the marginal effect of ULT in the treated cohort using 1:1 propensity score-matched cohorts in which the characteristics at enrollment were balanced. Third, we estimated the marginal effect in patients with asymptomatic hyperuricemia by excluding those with a history of gout. Fourth, we evaluated the association between ULT initiation and kidney outcomes, accounting for the competing risk of all-cause mortality.^40^ All analyses were conducted via R, version 4.4.3 (R Project for Statistical Computing). The target trial emulation analyses were performed via the R package “TrialEmulation” (version 0.0.3.9)^43^. Statistical significance was set as *P* < 0.05, and all tests were two-sided.

## Supplementary information

Supplements

STROBE checklist

## Data Availability

The data that support the findings of this study are available from the National Clinical Research Center for Kidney Disease and the Chinese Renal Disease Data System (CRDS) by emailing xux007@163.com or niesheng0202@126.com.
