# Supplementary material for: Urate-lowering therapy and kidney outcomes in patients with chronic kidney disease and hyperuricemia
Source: Signal Transduct Target Ther. 2025 Dec 9;10:399. doi: 10.1038/s41392-025-02497-0 (PMC12686408; doi:10.1038/s41392-025-02497-0)
Supplement: Supplementary file 1 — Supplements [file 41392_2025_2497_MOESM1_ESM.docx]

Supplementary Materials for

Urate-lowering therapy and kidney outcomes in patients with chronic kidney disease and hyperuricemia

Sheng Nie;^1#^ Shiyu Zhou;^1#^ Ruixuan Chen;^1#^ Lantian Li;^1^ Yinfang Sun;^1^ Jiao Liu;^1^ Luhua Jin;^1^ Xian Shao;^1^ Mingzhen Pang;^1^ Licong Su;^1^ Fan Luo;^1^ Xin Xu;^1*^ Fan Fan Hou^1*^

Correspondence to: Fan Fan Hou, MD, PhD, and Xin Xu, MD, PhD, Division of Nephrology, Nanfang Hospital, Southern Medical University, 1838 N Guangzhou Ave, Guangzhou 510515, China. Email: ffhouguangzhou@163.com or xux007@163.com

**This PDF file includes:**

Figures. S1

Tables S1 to S11


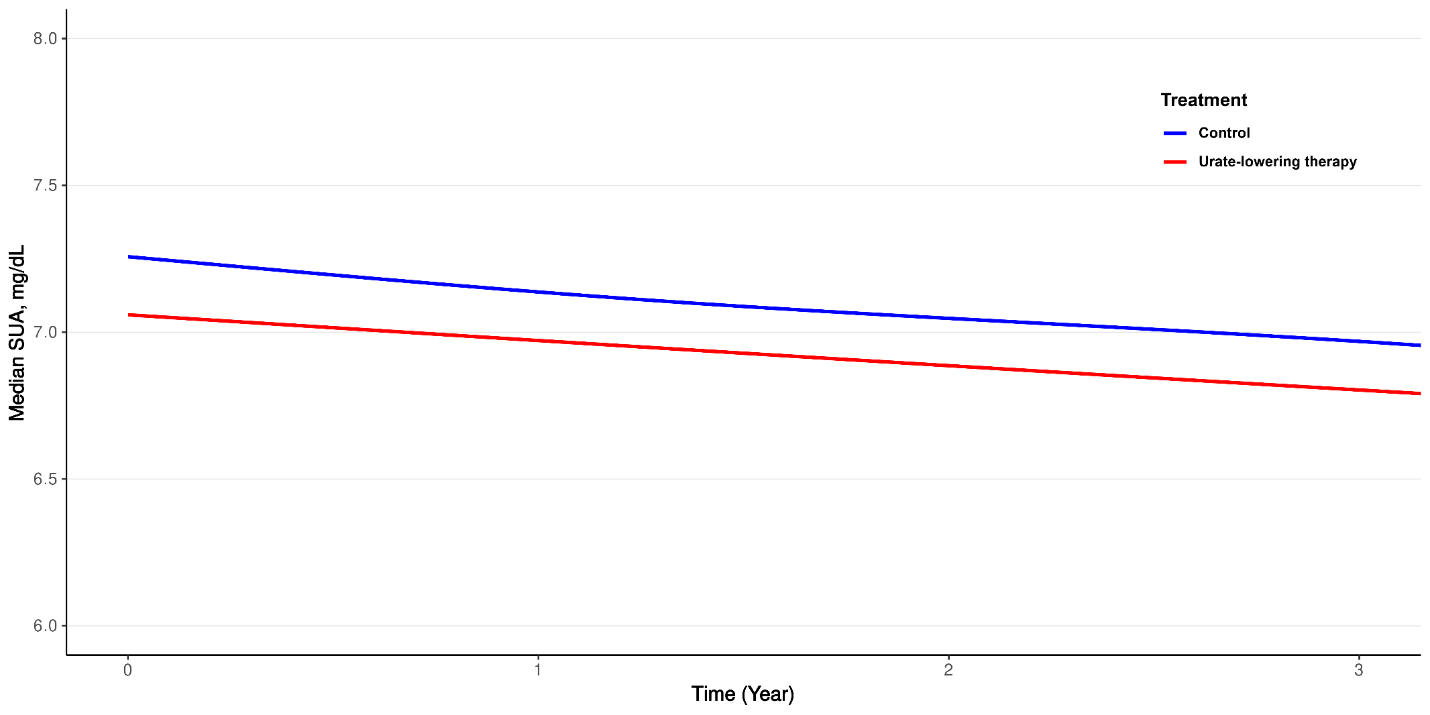
Figure. S1. Change in serum uric acid levels during follow-up in patients with urate-lowering therapy or not

Table S1. Specification and emulation of target trial

| **Protocol component** | **Target trial specification** | **Target trial emulation** |
| --- | --- | --- |
| Eligible criteria | Inclusion criteria:   - Adults (≥ 18 years old) with chronic kidney disease and hyperuricemia (serum uric acid > 7 mg/dL in men or 6 mg/dL in women) - At least 1 year of medication prescription records   Exclusion criteria:   - Suspected acute kidney injury - eGFR ≥ 60 or < 25 ml/min/1.73 m^2^ - Without receiving supportive care for chronic kidney disease - Dialysis or kidney transplant - Prior use of urate-lowering drugs within one-year | Same as for the target trials |
| Treatment strategy | Supportive care plus urate-lowering therapy versus supportive care alone (control) | Same as for the target trials |
| Treatment assignment | Subjects are randomly assigned to a treatment strategy and will be aware of the treatment strategy they are assigned to. | Each eligible participant was non-randomly assigned to a strategy with initiation of treatment. We assumed treatment strategy were exchangeable conditional on the baseline characteristics |
| Outcomes | Primary outcome is the composite of an eGFR decline >40% of baseline or end-stage kidney disease.  Secondary outcomes include end-stage kidney disease, all-cause mortality and cardiovascular mortality | Same as for the target trial |
| Follow-up | From baseline until the occurrence of composite kidney outcomes, death or the administrative end of follow-up (31 December 2022) | Same as for the target trial |
| Causal contrasts | Intention-to-treat effect  Per-protocol effect | Observational analogue to intention-to-treat |
| Statistical analysis | Intention-to-treat analysis  Per-protocol analysis: censored the patients when they deviated treatment strategy.  Subgroup analysis by age, sex, median baseline serum uric acid, baseline eGFR, median Charlson’s comorbidity score, urine albumin–creatinine ratio, present of hypertension, diabetes, cancer, gout, ischemic heart disease, kidney stone, use of renin angiotensin system inhibitors, statins, and sodium–glucose cotransporter 2 inhibitors. | Same as for the target trials, where the intention-to-treat analysis and per-protocol analysis with conducted via sequence trial emulation. We use pooled logistic regression analysis to estimate the effect of urate-lowering therapy via comparison of 3-year risk of study outcomes expressed as risk differences and standardized survival curves. Time-varying stabilized inverse-probability weights will be used to adjust for time-varying confounding associated with outcome of interest.  For intention-to-treat analyses we fitted a pooled logistic regression with an indicator for of assigned strategy. The inverse probability of censoring weights was calculated to account for censoring of follow-up.  For per-protocol analyses we fitted a model after censoring participants when they deviate from assigned treatment. The inverse probability of treatment and censoring weights were calculated to handle treatment switching and dependent censoring. |

Table S2. Summary of emulated trials among patients with urate-lowering therapy group versus control group

|  | **Total** | **Urate-lowering therapy** | **Control** |
| --- | --- | --- | --- |
| No. | 56936 | 11490 | 51944 |
| Person-trial, n | 269831 | 12357 | 257474 |
| Median number of person-trials (IQR) | 27(12-48) | 21(6-41) | 27(12-48) |
| Primary outcome, n | 5383 | 927 | 4456 |
| Death, n | 3215 | 185 | 3030 |
| Median follow-up (IQR), days | 783(347-1433) | 579(145-1208) | 793(355-1445) |

**Table S3. Three-year cumulative incidences and the risk differences among patients with urate-lowering therapy group versus control group in the per-protocol analysis**

| **Study outcomes** | **No. of events/100 PY** | **Cumulative incidence, % (95%CI)** | **Risk difference, % (95%CI)** |
| --- | --- | --- | --- |
| **Primary outcome^*^** |  |  |  |
| Control | 6.5 | 22.84 (19.49 to 28.99) | Reference |
| Urate-lowering therapy | 6.4 | 16.62 (15.69 to 19.17) | -6.22 (-10.51 to -2.10) |
| **Secondary outcomes** |  |  |  |
| **End-stage kidney disease** |  |  |  |
| Control | 4.4 | 12.82 (12.11 to 13.76) | Reference |
| Urate-lowering therapy | 3.7 | 12.73 (11.95 to 13.73) | -0.09 (-1.31 to 1.25) |
| **All-cause mortality** |  |  |  |
| Control | 4.9 | 18.75 (18.40 to 19.20) | Reference |
| Urate-lowering therapy | 7.1 | 15.50 (14.92 to 16.18) | -3.25 (-4.09 to -2.58) |
| **Cardiovascular mortality** |  |  |  |
| Control | 2.5 | 8.39 (8.12 to 8.74) | Reference |
| Urate-lowering therapy | 3.0 | 7.45 (7.03 to 8.00) | -0.95 (-1.59 to -0.42) |

Abbreviation: PY, person-year

^*^Primary outcome was a composite of a 40% eGFR decline from baseline or end-stage kidney disease. **Table S4. Three-year cumulative incidences of safety and negative control outcomes and the risk differences for urate-lowering therapy versus control group.**

| **Outcomes** | **No. of events/1000 PY** | **Cumulative incidence, % (95%CI)** | **Risk difference, % (95%CI)** |
| --- | --- | --- | --- |
| **Safety outcome*** |  |  |  |
| Control | 0.6 | 1.44 (1.35 to 1.56) | Reference |
| Urate-lowering therapy | 0.8 | 1.51 (1.23 to 1.92) | 0.07 (-0.24 to 0.41) |
| **Negative control outcome^#^** |  |  |  |
| Control | 4.1 | 9.49 (9.29 to 9.71) | Reference |
| Urate-lowering therapy | 6.9 | 10.05 (9.34 to 10.76) | 0.56 (-0.13 to 1.22) |

Abbreviation: PY, person-year

^*^ safety outcome was defined as the *International Classification of Diseases, 10th Revision* code, including cutaneous reactions (L27.0-L27.1, L27.8-L27.9, T78.4, L50.0-L50.3, L50.8-L50.9, L51.0-L51.3, L51.8-L51.9, D72.12, L08.0, T88.7, T50.905A, R21, M33.1), hypersensitivity (T50.400), or hepatotoxicity (K71).

^*^ Negative control outcome was defined as the *International Classification of Diseases, 10th Revision* code, including gastritis and duodenitis (K29).

**Table S5. Three-year cumulative incidences and the risk differences of primary outcome for each of three urate-lowering drugs versus control group.**

| **Primary outcome^*^** | **No. of events/100 PY** | **Cumulative incidence, % (95%CI)** | **Risk difference, % (95%CI)** |
| --- | --- | --- | --- |
| **Allopurinol versus control** |  |  |  |
| Control | 5.6 | 16.13 (14.75 to 19.04) | Reference |
| Urate-lowering therapy | 4.8 | 13.53 (11.67 to 16.27) | -2.61 (-5.13 to -0.14) |
| **Febuxostat versus control** |  |  |  |
| Control | 6.3 | 21.92 (21.20 to 23.00) | Reference |
| Urate-lowering therapy | 8.2 | 20.28 (18.04 to 23.22) | -1.64 (-4.02 to 0.86) |
| **Benzbromarone versus control** |  |  |  |
| Control | 5.6 | 17.21 (15.09 to 22.52) | Reference |
| Urate-lowering therapy | 4.5 | 13.42 (11.84 to 17.15) | -3.79 (-7.12 to -1.60) |

Abbreviation: PY, person-year

^*^ Primary outcome was a composite of a 40% eGFR decline from baseline or end-stage kidney disease.

**Table S6. The baseline characteristics for person-trials in the emulated target trial after propensity score matching**

| **Characteristics** | **Total**  **(27,032 person-trials)** | **Urate-lowering therapy group**  **(13,516 person-trials)** | **Control group**  **(13,516 person-trials)** | **Standardized Difference*** |
| --- | --- | --- | --- | --- |
| Age, yr | 70 (59-79) | 70 (59-78) | 70 (59-79) | 0.023 |
| Sex, Male (%) | 17056 (63.1) | 8529 (63.1) | 8527 (63.1) | <0.001 |
| Hospitalization (%) | 15885 (58.8) | 7600 (56.2) | 8285 (61.3) | 0.103 |
| Number of hospitalizations in the year | 1 (0-1) | 1 (0-1) | 1 (0-1) | 0.010 |
| Intensive care unit (%) | 523 (1.9) | 264 (2.0) | 259 (1.9) | 0.003 |
| Number of intensive care unit in the year | 0 (0-0) | 0 (0-0) | 0 (0-0) | 0.006 |
| Body-mass index, kg/m^2^ | 21.9 (21.2-25.2) | 21.9 (21.2-25.3) | 21.9 (21.1-25.2) | 0.025 |
| SBP, mmHg | 132 (120-147) | 132 (120-147) | 133 (120-148) | 0.021 |
| DBP, mmHg | 77 (69-85) | 76.5 (69-86) | 77 (69-85) | 0.002 |
| Surgery (%) | 2811 (10.4) | 1341 (9.9) | 1470 (10.9) | 0.031 |
| **Laboratory parameters** |  |  |  |  |
| Serum uric acid, mg/dL | 9.3 (8.2-10.7) | 9.4 (8.4-10.7) | 9.2 (8.1-10.7) | 0.047 |
| Serum creatinine, μmol/L | 131.3 (113.6-155.6) | 131.8 (114-156) | 131 (113-155) | 0.018 |
| Number of serum creatinine tests within 6 months | 2 (1-4) | 2 (1-4) | 2 (1-4) | 0.077 |
| eGFR, ml/min/1.73m^2^ | 43.7 (35.3-51.6) | 43.6 (35.3-51.5) | 43.9 (35.4-51.7) | 0.013 |
| <30 | 2981 (11.0) | 1492 (11.0) | 1489 (11.0) |  |
| 30-44 | 11607 (42.9) | 5879 (43.5) | 5728 (42.4) |  |
| ≥45 | 12444 (46.0) | 6145 (45.5) | 6299 (46.6) |  |
| UACR, mg/g | 16 (11-198) | 16 (11-200) | 16 (11-179) | <0.001 |
| <30 | 13042 (48.2) | 6653 (49.2) | 6389 (47.3) |  |
| 30-299 | 5399 (20.0) | 2697 (20.0) | 2702 (20.0) |  |
| ≥300 | 3937 (14.6) | 2081 (15.4) | 1856 (13.7) |  |
| Unknown | 4654 (17.2) | 2085 (15.4) | 2569 (19.0) |  |
| Triglyceride, mmol/L | 1.5 (1-2.2) | 1.5 (1-2.2) | 1.4 (1-2.1) | 0.046 |
| Total cholesterol, mmol/L | 4.4 (3.6-5.3) | 4.4 (3.6-5.4) | 4.4 (3.6-5.3) | 0.020 |
| LDL-C, mmol/L | 2.5 (1.9-3.2) | 2.5 (1.9-3.2) | 2.5 (1.9-3.2) | 0.011 |
| Serum albumin, g/L | 40.2 (36.4-43.7) | 40.2 (36.5-43.7) | 40.2 (36.4-43.7) | 0.016 |
| Hemoglobin, g/L | 125 (109-139) | 124 (109-139) | 125 (109-140) | 0.004 |
| Glycated hemoglobin level, % | 6.2 (5.7-7.2) | 6.3 (5.7-7.2) | 6.2 (5.7-7.2) | 0.006 |
| **Co-morbidity (%)** |  |  |  |  |
| Charlson score | 5 (4-7) | 5 (3-7) | 5 (4-7) | 0.015 |
| Gout | 4911 (18.2) | 2598 (19.2) | 2313 (17.1) | 0.055 |
| Kidney stone | 4961 (18.4) | 2507 (18.5) | 2454 (18.2) | 0.01 |
| Hypertension | 15279 (56.5) | 7779 (57.6) | 7500 (55.5) | 0.042 |
| Diabetes | 7363 (27.2) | 3729 (27.6) | 3634 (26.9) | 0.016 |
| Myocardial infarction | 1476 (5.5) | 739 (5.5) | 737 (5.5) | 0.001 |
| Ischemic heart disease | 7144 (26.4) | 3619 (26.8) | 3525 (26.1) | 0.016 |
| PVD | 5620 (20.8) | 2772 (20.5) | 2848 (21.1) | 0.014 |
| Stroke | 5825 (21.5) | 2898 (21.4) | 2927 (21.7) | 0.005 |
| Heart failure | 6009 (22.2) | 3052 (22.6) | 2957 (21.9) | 0.017 |
| Cancer | 2674 (9.9) | 1354 (10.0) | 1320 (9.8) | 0.008 |
| Proteinuria | 10859 (40.2) | 5484 (40.6) | 5375 (39.8) | 0.016 |
| **Co-medications (%)** |  |  |  |  |
| Number of medications in 3 months | 12 (5-19) | 12 (6-18) | 13 (5-19) | 0.012 |
| RASi | 16084 (59.5) | 8117 (60.1) | 7967 (58.9) | 0.023 |
| Statins | 15090 (55.8) | 7547 (55.8) | 7543 (55.8) | 0.001 |
| Diuretics | 13628 (50.4) | 6790 (50.2) | 6838 (50.6) | 0.007 |
| Calcium channel blockers | 14783 (54.7) | 7390 (54.7) | 7393 (54.7) | <0.001 |
| SGLT2 inhibitors | 735 (2.7) | 376 (2.8) | 359 (2.7) | 0.008 |

* parameters with an SMD higher than 0.1 were regarded as unbalanced between the groups

IQR, interquartile range; DBP, Diastolic blood pressure; LDL-C, Low-density lipoprotein cholesterol; SBP, Systolic blood pressure; PVD, Peripheral vascular disease; SGLT-2 Sodium–glucose cotransporter 2; RASI, Renin Angiotensin System inhibitor; UACR, Urine albumin–creatinine ratio

**Table S7. Three-year cumulative incidences and risk differences among patients with urate-lowering therapy versus control group after propensity score matching**

| **Study outcomes** | **No. of events/100 PY** | **Cumulative incidence, % (95%CI)** | **Risk difference, % (95%CI)** |
| --- | --- | --- | --- |
| **Primary outcome^*^** |  |  |  |
| Control | 6.2 | 19.49 (17.02 to 25.00) | Reference |
| Urate-lowering therapy | 6.9 | 18.3 (16.92 to 21.41) | -1.19 (-3.80 to 0.99) |
| **Secondary outcomes** |  |  |  |
| **End-stage kidney disease** |  |  |  |
| Control | 3.8 | 11.98 (10.59 to 13.29) | Reference |
| Urate-lowering therapy | 4.2 | 11.09 (9.99 to 12.45) | -0.89 (-1.89 to 0.23) |
| **All-cause mortality** |  |  |  |
| Control | 5.6 | 15.64 (14.91 to 16.39) | Reference |
| Urate-lowering therapy | 6.0 | 14.06 (13.19 to 15.13) | -1.58 (-2.41 to -0.79) |
| **Cardiovascular mortality** |  |  |  |
| Control | 1 2.8 | 8.24 (8.01 to 8.54) | Reference |
| Urate-lowering therapy | 2 3.1 | 7.46 (6.99 to 8.07) | -0.78 (-1.25 to -0.27) |

Abbreviation: PY, person-year

^*^ Primary outcome was a composite of a 40% eGFR decline from baseline or end-stage kidney disease.

**Table S8. Three-year cumulative incidences and the risk differences for urate-lowering therapy versus control group accounting for the competing risk of all-cause death.**

| **Study outcomes** | **No. of events/100 PY** | **Cumulative incidence, % (95%CI)** | **Risk difference, % (95%CI)** |
| --- | --- | --- | --- |
| **Intention-to-treat** |  |  |  |
| **Primary outcome*** |  |  |  |
| Control | 8.9 | 22.28 (20.77 to 24.81) | Reference |
| Urate-lowering therapy | 9.6 | 19.09 (17.70 to 20.79) | -3.19 (-4.97 to -0.76) |
| **Secondary outcomes** |  |  |  |
| **End-stage kidney disease** |  |  |  |
| Control | 6.9 | 10.69 (10.06 to 11.48) | Reference |
| Urate-lowering therapy | 7.3 | 9.73 (8.63 to 10.67) | -0.96 (-2.10 to 0.11) |
|  |  |  |  |
| **Per-protocol** |  |  |  |
| **Primary outcome*** |  |  |  |
| Control | 8.2 | 24.39 (21.37 to 28.61) | Reference |
| Urate-lowering therapy | 9.3 | 16.98 (15.98 to 18.73) | -7.41 (-10.88 to -3.39) |
| **Secondary outcomes** |  |  |  |
| **End-stage kidney disease** |  |  |  |
| Control | 4.4 | 9.24 (8.75 to 9.93) | Reference |
| Urate-lowering therapy | 3.7 | 9.18 (8.55 to 9.97) | -0.06 (-0.97 to 0.76) |

Abbreviation: PY, person-year

^*^ Primary outcome was a composite of a 40% eGFR decline from baseline or end-stage kidney disease.

**Table S9. The baseline characteristics for person-trials with asymptomatic hyperuricemia in the emulated target trial**

| **Characteristics** | **Total**  **(244,349 person-trials)** | **Urate-lowering therapy group**  **(9904 person-trials)** | **Control group**  **(234,445 person-trials)** |
| --- | --- | --- | --- |
| Age, yr | 71 (60-80) | 70 (59-78) | 71 (60-80) |
| Sex, Male (%) | 123173 (50.4) | 5874 (59.3) | 117299 (50.0) |
| Hospitalization (%) | 72734 (29.8) | 5689 (57.4) | 67045 (28.6) |
| Number of hospitalizations in the year | 1 (0-2) | 1 (0-1) | 1 (0-2) |
| Intensive care unit (%) | 1962 (0.8) | 154 (1.6) | 1808 (0.8) |
| Number of Intensive care unit in the year | 0 (0-0) | 0 (0-0) | 0 (0-0) |
| Body-mass index, kg/m^2^ | 21.9 (21.0-25.0) | 21.9 (21.1-25.2) | 21.9 (21.0-25.0) |
| SBP, mmHg | 131 (120-145) | 133 (120-147) | 131 (120-145) |
| DBP, mmHg | 76 (68-83) | 77 (69-86) | 76 (68-83) |
| Surgery (%) | 28034 (11.5) | 1003 (10.1) | 27031 (11.5) |
| **Laboratory parameters** |  |  |  |
| Serum uric acid, mg/dL | 8.0 (7.2-9.1) | 9.3 (8.3-10.6) | 8 (7.2-9) |
| Serum creatinine, μmol/L | 122 (106-145) | 131 (113-156) | 122 (105-144) |
| Number of serum creatinine tests within 6 months | 2 (1-4) | 2 (1-4) | 2 (1-4) |
| eGFR, ml/min/1.73m^2^ | 46 (37.7-53.1) | 43.2 (35.1-51.4) | 46.1 (37.8-53.1) |
| <30 | 20094 (8.2) | 1127 (11.4) | 18967 (8.1) |
| 30-44 | 93588 (38.3) | 4352 (43.9) | 89236 (38.1) |
| ≥45 | 130667 (53.5) | 4425 (44.7) | 126242 (53.8) |
| UACR, mg/g | 17 (11-200) | 17 (11-217) | 17 (11-200) |
| <30 | 108537 (44.4) | 4742 (47.9) | 103795 (44.3) |
| 30-299 | 47313 (19.4) | 2009 (20.3) | 45304 (19.3) |
| ≥300 | 37089 (15.2) | 1724 (17.4) | 35365 (15.1) |
| Unknown | 51410 (21.0) | 1429 (14.4) | 49981 (21.3) |
| Triglyceride, mmol/L | 1.4 (1-2.1) | 1.5 (1.1-2.3) | 1.4 (1-2.1) |
| Total cholesterol, mmol/L | 4.5 (3.7-5.4) | 4.5 (3.6-5.4) | 4.5 (3.7-5.4) |
| LDL-C, mmol/L | 2.5 (1.9-3.3) | 2.5 (1.9-3.3) | 2.5 (1.9-3.3) |
| Serum albumin, g/L | 40.3 (36.4-43.7) | 40.2 (36.6-43.6) | 40.3 (36.4-43.7) |
| Hemoglobin, g/L | 122 (107-136) | 124 (109-138.3) | 122 (107-136) |
| Glycated hemoglobin level, % | 6.4 (5.8-7.5) | 6.3 (5.8-7.2) | 6.4 (5.8-7.5) |
| **Co-morbidity (%)** |  |  |  |
| Charlson score | 6 (4-7) | 5 (3-7) | 6 (4-7) |
| Gout | 0 (0) | 0 (0) | 0 (0) |
| Kidney stone | 45327 (18.6) | 1574 (15.9) | 43753 (18.7) |
| Hypertension | 151740 (62.1) | 5684 (57.4) | 146056 (62.3) |
| Diabetes | 81620 (33.4) | 2779 (28.1) | 78841 (33.6) |
| Myocardial infarction | 14304 (5.9) | 504 (5.1) | 13800 (5.9) |
| Ischemic heart disease | 70832 (29.0) | 2565 (25.9) | 68267 (29.1) |
| PVD | 58714 (24.0) | 1985 (20.0) | 56729 (24.2) |
| Stroke | 58209 (23.8) | 2086 (21.1) | 56123 (23.9) |
| Heart failure | 55783 (22.8) | 2178 (22.0) | 53605 (22.9) |
| Cancer | 44653 (18.3) | 976 (9.9) | 43677 (18.6) |
| Proteinuria | 105192 (43.0) | 4325 (43.7) | 100867 (43.0) |
| **Co-medications (%)** |  |  |  |
| Number of medications in 3 months | 10 (3-17) | 12 (6-18) | 9 (3-17) |
| RASi | 144911 (59.3) | 6602 (66.7) | 138309 (59.0) |
| Statins | 132019 (54.0) | 5942 (60.0) | 126077 (53.8) |
| Diuretics | 124839 (51.1) | 5439 (54.9) | 119400 (50.9) |
| Calcium channel blockers | 137756 (56.4) | 5848 (59.0) | 131908 (56.3) |
| SGLT2 inhibitors | 5184 (2.1) | 331 (3.3) | 4853 (2.1) |

IQR, interquartile range; DBP, Diastolic blood pressure; LDL-C, Low-density lipoprotein cholesterol; SBP, Systolic blood pressure; PVD, Peripheral vascular disease; SGLT-2 Sodium–glucose cotransporter 2; RASI, Renin Angiotensin System inhibitor; UACR, Urine albumin–creatinine ratio

**Table S10.** **Three-year cumulative incidences and the risk differences among patients with asymptomatic hyperuricemia for urate-lowering therapy versus control group.**

| **Study outcomes** | **No. of events/100 PY** | **Cumulative incidence, % (95%CI)** | **Risk difference, % (95%CI)** |
| --- | --- | --- | --- |
| **Primary outcome^*^** |  |  |  |
| Control | 6.5 | 22.47 (21.47 to 24.42) | Reference |
| Urate-lowering therapy | 7.2 | 18.47 (16.76 to 21.25) | -4.00 (-5.51 to -2.76) |
| **Secondary outcomes** |  |  |  |
| **End-stage kidney disease** |  |  |  |
| Control | 3.9 | 12.94 (11.81 to 14.54) | Reference |
| Urate-lowering therapy | 4.3 | 11.42 (10.33 to 13.11) | -1.53 (-2.73 to -0.22) |
| **All-cause mortality** |  |  |  |
| Control | 5.6 | 15.64 (14.91 to 16.39) | Reference |
| Urate-lowering therapy | 6.0 | 14.06 (13.19 to 15.13) | -1.58 (-2.41 to -0.79) |
| **Cardiovascular mortality** |  |  |  |
| Control | 2.8 | 8.24 (8.01 to 8.54) | Reference |
| Urate-lowering therapy | 3.1 | 7.46 (6.99 to 8.07) | -0.78 (-1.25 to -0.27) |

Abbreviation: PY, person-year

^*^ Primary outcome was a composite of a 40% eGFR decline from baseline or end-stage kidney disease.

**Table S11. Definitions of comorbidities**

| **Variables** | **ICD-10 codes** |
| --- | --- |
| Gout | M10 |
| Kidney stone | N20.0 |
| Hypertension | I10-I15 |
| Diabetes | E10-E14 |
| Myocardial infarction | I21 |
| Ischemic heart disease | I20-I25 |
| PVD | I70, I72, I73.1, I73.9, I74, I77.1 |
| Stroke | I61, I62, I63, I65, I66, I67 |
| Heart failure | I11.0, I13.0, I13.2, I50 |
| Cancer | C00-D09 |
| Proteinuria | Defined as urine albumin–creatinine ratio > 30 mg/g before the index date |
| **Charlson Comorbidity Index** |  |
| Cerebrovascular disease | G45, G46, H34.0, I60–I69 |
| Dementia | F00–F03, F05.1, G30, G31.1 |
| Chronic pulmonary disease | I27.8, I27.9, J40–J47, J60–J67, J68.4, J70.1, J70.3 |
| Rheumatic disease | M05, M06, M31.5, M32–M34, M35.1, M35.3, M36.0 |
| Peptic ulcer disease | K25-K28 |
| Mild liver disease | B18, K70.0–K70.3, K70.9, K71.3–K71.5, K71.7, K73, K74, K76.0, K76.2–76.4, K76.8, K76.9, Z94.4 |
| Diabetes without chronic complication | E10.0, E10.1, E10.6, E10.8, E10.9, E11.0, E11.1, E11.6, E11.8, E11.9, E12.0, E12.1, E12.6, E12.8, E12.9, E13.0, E13.1, E13.6, E13.8, E13.9, E14.0, E14.1, E14.6, E14.8, E14.9 |
| Diabetes with chronic complication | E10.2–E10.5, E10.7, E11.2–E11.5, E11.7, E12.2–E12.5, E12.7, E13.2–E13.5, E13.7, E14.2–E14.5, E14.7 |
| Hemiplegia or paraplegia | G04.1, G11.4, G80.1, G80.2, G81, G82, G83.0–G83.4, G83.9 |
| Renal disease | I12.0, I13.1, N03.2–N03.7, N05.2–N05.7, N18, N19, N25.0, Z49.0–Z49.2, Z94.0, Z99.2 |
| Malignancy | C00–C26, C30–C34, C37–C41, C43, C45–C58, C60–C76, C81–C85, C88, C90–C97 |
| Moderate or severe liver disease | I85.0, I85.9, I86.4, I98.2, K70.4, K71.1, K72.1, K72.9, K76.5, K76.6, K76.7 |
| Metastatic solid tumor | C77–C80 |
| AIDS/HIV | B20–B22, B24 |

AIDS, acquired immune deficiency syndrome; HIV, human immunodeficiency virus; PVD, peripheral vascular disease
